# NMDAR and JNK Activation in the Spinal Trigeminal Nucleus Caudalis Contributes to Masseter Hyperalgesia Induced by Stress

**DOI:** 10.3389/fncel.2019.00495

**Published:** 2019-11-14

**Authors:** Wenqing Lin, Yajuan Zhao, Baixiang Cheng, Haidan Zhao, Li Miao, Qiang Li, Yongjin Chen, Min Zhang

**Affiliations:** ^1^State Key Laboratory of Military Stomatology, Department of General Dentistry and Emergency, National Clinical Research Center for Oral Diseases, Shaanxi International Joint Research Center for Oral Diseases, School of Stomatology, Fourth Military Medical University, Xi’an, China; ^2^Department of Stomatology, Air Force Medical Center, Beijing, China

**Keywords:** psychological stress, chronic masseter pain, spinal trigeminal nucleus caudalis, N-methyl-D-aspartate receptor, c-Jun N- terminal kinase, intercellular communication

## Abstract

It is commonly accepted that psychological stress is closely associated with the occurrence and development of chronic orofacial pain. However, the pathogenesis underlying this process has not been fully elucidated. In the present study, we explored the role of N-methyl-D-aspartate receptors (NMDARs) and Jun N-terminal kinase (JNK) mediated intercellular communication between neurons and astrocytes in the spinal trigeminal nucleus caudalis (Vc) in the induction of masseter hyperalgesia by psychological stress in rats. We found that subjecting rats to 14 days of restraint stress (8 h/d) caused a significant decrease in body weight gain, behavioral changes and marked masseter hyperalgesia in the rats. We also found that exposure to restraint stress for 14 days caused the expression of pJNK in astrocytes in the Vc to significantly increase, and intrathecally infusing a JNK inhibitor significantly prevented restraint stress-induced masseter hyperalgesia in the rats. In addition, after exposure to restraint stress for 14 days, the stressed group exhibited a noticeably increased expression level of pNR2B in neurons in the Vc. Then, we intrathecally injected MK-801 (an NMDAR inhibitor) and ifenprodil (a selective NR2B subunit antagonist) and observed that the two types of inhibitors not only alleviated masseter hyperalgesia but also significantly inhibited the phosphorylation of JNK in the Vc after restraint stress; this indicates that the effect of NMDAR antagonists may influence the activation of astrocytic JNK. Furthermore, inhibitors of neuronal nitric oxide synthase (nNOS) activation and guanylate cyclase (GC) inhibitor could not only inhibit the expression of pJNK in the Vc, but also effectively alleviate masseter hyperalgesia induced by restraint stress. Taken together, our results suggest that NMDAR activation could increase JNK phosphorylation in astrocytes after restraint stress, which may depend on the nNOS-GC pathway. The intercellular communication between neurons and astrocytes in the Vc may play a key role in the induction of masseter muscle hyperalgesia by psychological stress in rats.

## Introduction

With the development of modern society and the formation of an increasingly fast-paced lifestyle, increasing attention has been drawn to the importance of psychological factors in people’s health. Many studies have proven that psychological stress is related to the occurrence and development of a variety of systemic diseases (Luppino et al., 2011; de Brouwer et al., 2013; Lee et al., 2015). Presently, the researches on the influence of psychological stress on the oral and maxillofacial regions are clear; psychological stress can aggravate the occurrence and development of periodontitis (Zhao et al., 2012), delay the healing of oral mucosal diseases (Horan et al., 2005) and increase the tension of masticatory muscles (Song et al., 2014). However, as one of the most common oral and maxillofacial diseases, chronic orofacial pain, because of its complex etiology, is affected by many factors. Thus, far, the underlying cellular and molecular mechanisms are primarily unknown.

In the maxillofacial pain pathway, the primary afferent neurons are located in the trigeminal ganglion, whose peripheral terminals and central terminals are distributed in the face and spinal trigeminal nucleus, respectively. Many studies have suggested that the spinal trigeminal nucleus caudalis (Vc) plays a vital role in the transmission and regulation of orofacial nociceptive information (Guo et al., 2010; Wang et al., 2015). Based on this hypothesis, a series of studies have been carried out by our research group. Astrocytes in the Vc have been confirmed as key players in the induction and maintenance of masseter hyperalgesia by psychological stress in rats (Zhao et al., 2015). Astrocytes cannot generate action potentials themselves; however, the synapses of neurons are tightly wrapped by glial cells. Currently, some scholars believe that glial cells are part of the synaptic structure of neurons and participate in neural information transmission (Ren, 2010). Studies (Guo et al., 2010; Park et al., 2011; Yang et al., 2018) have indicated that the neurons in the Vc are activated following orofacial nociceptive stimulation, and N-methyl-D-aspartate (NMDA), a glutamate neurotransmitter, is involved in signal transmission between astrocytes and neurons, which plays an important role in synaptic transmission. However, it is not clear how neurons mediate the activation of astrocytes through NMDA and its receptors (NMDARs) in chronic orofacial pain.

Recent studies have shown that c-Jun N-terminal kinase (JNK), a member of the mitogen-activated protein kinase (MAPK) family, is upregulated significantly in the dorsal horn of the spinal cord after nerve injury and is specifically distributed in astrocytes (Yamamoto et al., 2013). Thus, far, JNK activation is used as a reliable indicator of the pain-related activation of astrocytes (Ji et al., 2009). Studies have also demonstrated that NMDA receptors play an important role in the phosphorylation of JNK in cultured cortical neurons (Centeno et al., 2007). In addition, studies have found that the NMDA-dependent activation of JNK is involved in morphine tolerance (Sanna et al., 2015). However, it is not clear whether NMDAR activates astrocytic JNK under chronic orofacial pain conditions. In the present study, we tested our hypothesis that neuronal NMDAR activation is involved in the phosphorylation of astrocytic JNK in the Vc, which is involved in masseter hyperalgesia induced by psychological stress, through reciprocal crosstalk.

## Materials and Methods

### Experimental Animals and Model Establishment

All experiments were performed under the approval of the Animal Use and Care Committee for Research and Education of the Fourth Military Medical University (Xi’an, China). Adult male Sprague–Dawley rats (180–220 g) were used in this study. The rats were divided into the control group, and 1-day, 3-days, 5-days, 7-days, 9-days, 11-days and 14-days stress groups (*n* = 8). The animal model was established by subjecting rats to restraint stress. The animals were kept in a restrainer for 8 h/day. The restrainers were made of inflexible wire mesh so that the bodies of the animals were not constricted (Imbe et al., 2004). This kind of restraint stress minimized the physical pressure on the animals. The rats were not allowed to eat or drink during the stress procedure.

### Behavioral Testing

The open-field test and elevated plus maze test (RD 1412-OF, Shanghai Mobile Datum Corporation, Shanghai, China) were used to evaluate the animal model. The activity of each rat was automatically monitored using a digital video camera (McLean et al., 2010). According to our previous study (Zhao et al., 2015), masseter muscle mechanical sensitivity was evaluated in the animals by an electronic von Frey anesthesiometer (IITC Life Science Instruments, Woodland Hills, CA, USA). In our study, force was applied 10 mm inferior to the central point of the line between the orbit and the tragus with the probe.

### Intrathecal Implantation and Drug Administration

The methods used for intrathecal implantation and drug administration were based on previous studies (Zhao et al., 2015). Under pentobarbital anesthesia (50 mg/kg, i.p.), an incision was made in the midline of the rats’ back at the level of the thoracic vertebrae. Polyethylene (PE) tubing (I.D. 0.28 mm and O.D. 0.61 mm) was passed caudally from the T8 level to the L3 level of the spinal cord of the tube end was left exposed free for 2 cm. After surgery, the animals recovered for 7 days. Only the rats that fully recovered were used for further experiments.

Detailed information about the drugs used is shown in Table 1. Normal saline (0.9%) was used as the negative control in the experiments. For the continuous intrathecal infusion of SP600125, the catheter was connected to an osmotic pump (11-days pump, 1 μl/h; Durect, Cupertino, CA, USA), which was placed subcutaneously on the back of the rats and was filled with SP600125 (1 μg/μl) or 0.9% saline. MK801 (100 μg/kg), ifenprodil (100 μg/kg), NMDA (1 μg/kg), 7-NINA (100 μg/kg), ODQ (100 μg/kg) and 0.9% saline were intrathecally injected *via* the catheter twice a day (before stress and after stress).

**Table 1:** Drugs used in the study.

Drugs	Mechanisms of action	Solvent	Source
SP600125	A selective inhibitor of c-Jun N-terminal kinase	1% DMSO	Calbiochem
MK-801	A non-competitive NMDA receptor antagonist	saline	Sigma
Ifenprodil	A selective antagonist of NMDA receptors containing the NR2B subunit	1% DMSO	Sigma
NMDA	A specific agonist of the NMDA receptor	1% DMSO	Sigma
7-NINA	A selective neuronal nitric oxide synthase inhibitor	20% DMSO	Sigma
ODQ	A selective inhibitor of the nitric oxide sensitive guanylyl cyclase	20% DMSO	Sigma

### Immunofluorescence Staining

The rats were anesthetized with pentobarbital (60 mg/kg) and were perfused with 100 ml of 0.9% saline followed by 500 ml of 0.1 M phosphate buffer (PB, pH 7.3) containing 4% paraformaldehyde and 2% picric acid. The medulla and upper cervical spinal cord were removed, and transverse frozen sections (30 μm thick) of the caudal medulla were cut by a cryostat microtome (Leica CM1800; Heidelberg, Germany), and the sections were collected in 0.01 M phosphate-buffered saline (PBS, pH 7.3). The sections were blocked with 2% donkey serum in 0.01 M PBS containing 0.3% Triton X-100 for 1 h at room temperature and then subjected to immunofluorescence staining. The sections were incubated overnight at room temperature with a rabbit anti-NR2B antibody (1:100; Santa Cruz) mixed with a mouse anti-NeuN (1:3,000; Millipore, Burlington, MA, USA) or mouse anti-GFAP (1:5,000; Millipore, Burlington, MA, USA) antibody or a rabbit anti-pJNK antibody (1:1,000; Cell Signaling) mixed with a mouse anti-glial fibrillary acidic protein (GFAP) antibody (1:5,000; Millipore, Burlington, MA, USA). After being rinsed, the sections were incubated for 4 h at room temperature with the Alexa Fluor 488-conjugated donkey anti-mouse IgG (1:500; Molecular Probes, Eugene, OR, USA) and an Alexa Fluor 594-conjugated donkey anti-rabbit IgG (1:800; Molecular Probes, Eugene, OR, USA) secondary antibodies. A confocal laser microscope (FV1000; Olympus, Tokyo, Japan) was used to image the immunofluorescence staining for each group. For semi-quantification, images of the same area of the superficial laminae of the Vc were captured. An Image-Pro Plus system (Media Cybernetics, Rockville, MD, USA) was used to measure the signal intensity, and the optical density of each section was determined.

### Western Blotting

The rats were anesthetized with pentobarbital (60 mg/kg) and rapidly sacrificed on dry ice. The Vc region was quickly dissected and placed in a 1.5-ml centrifuge tube. The selected region was homogenized, and the homogenates were centrifuged at 12,000 rpm for 10 min. The supernatants were heated at 100°C for 5 min and cooled. Then, the protein concentration of the supernatant was evaluated by a BCA Protein Assay Kit (Pierce, Rockford, IL, USA). The samples for electrophoresis were loaded onto 10% SDS-poly-acrylamide gels with standard Laemmli solutions (Bio-Rad Laboratories, Hercules, CA, USA), and the proteins of interest were transferred onto a polyvinylidene difluoride (PVDF) membrane (Immobilon-P, Millipore, Billerica, MA, USA). The membranes were placed in a blocking solution (Tris-buffered saline with 0.02% Tween-20 and 3% nonfat dry milk powder) for 1 h and then incubated overnight with rabbit anti-JNK (1:1,000; Cell Signaling), rabbit anti-phosphorylated JNK (pJNK, 1:1,000; Cell Signaling Technology, Beverly, MA, USA), rabbit anti-phosphorylated NR2B (1:100; Santa Cruz Biotechnology, Santa Cruz, CA, USA) mouse anti-β-actin (1:50,000; Millipore, Burlington, MA, USA) primary antibodies. After being rinsed, the membranes were incubated for 2 h under gentle agitation with horseradish peroxidase (HRP)-conjugated secondary antibodies (anti-rabbit 1:3,000, anti-mouse 1:5,000; Amersham Pharmacia Biotech Inc., Piscataway, NJ, USA). An enhanced chemiluminescence detection method (Amersham) was used to detect the reaction, and Labworks Software (Ultra-Violet Products, Cambridge, UK) was used to analyze the densities of the protein blots. The levels of the target proteins normalized to β-actin levels and expressed as relative fold changes compared to the control or saline groups.

### Statistical Analysis

The experimental data are expressed as mean ± SD. All data were analyzed using SPSS version 19.0 software (SPSS Inc., Chicago, IL, USA). The body weight gain, behavioral tests, and von Frey test were evaluated using two-way ANOVA, and the Bonferroni correction was applied to adjust the *P*-value when the ANOVA indicated overall significance. The western blotting data in Figure 5 were analyzed by the *t*-test (two groups). The other Western blotting data were analyzed using one-way ANOVA followed by Dunnett’s *t*-test for multiple comparisons. *P* < 0.05 (double tail) was considered statistically significant in all cases.

**Figure 1:**
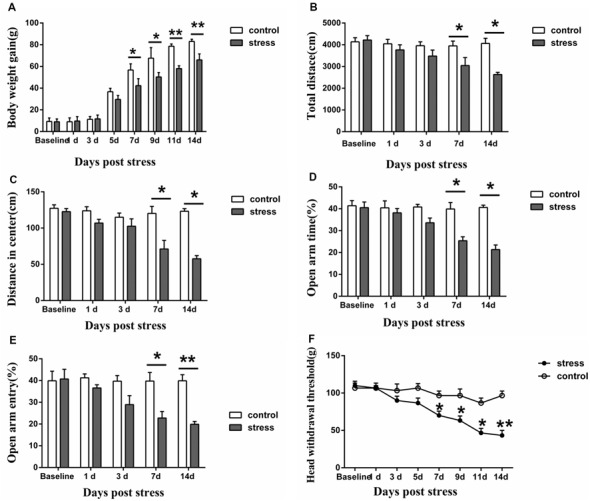
Effect of chronic restraint stress on body weight gain **(A)**, behavior **(B–E)** and the head withdrawal threshold **(F)** in rats. In the open field test, the total distance traveled **(B)** and the distance traveled in the center **(C)** were measured, and in the elevated plus-maze test, the percentage of open-arm entries **(D)** and the open-arm retention time **(E)** were measured. All data were calculated as the mean ± SD (*n* = 8/group). *P < 0.05, **P < 0.01 vs. the matched control group.

**Figure 2:**
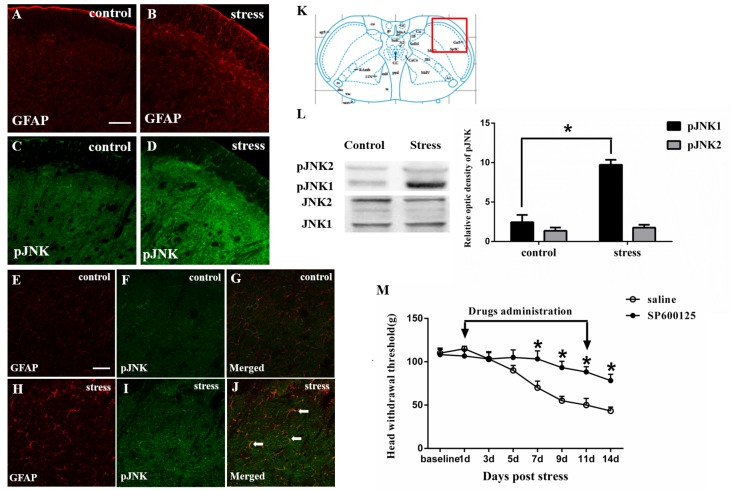
Restraint stress increases the expression of the astrocytic specific marker GFAP **(A,B)** and phosphorylated JNK (pJNK, **C,D**) in the Vc after exposure to restraint stress for 14 days. Panels **(E–J**) shows the higher magnification of double immunofluorescence in the control and stress group. The arrows indicate some typical cells double-labeled with pJNK and GFAP. Scale bars = 100 μm in **A** (applies to **A–D**) and 50 μm in **E** (applies to **E–J**). **(K)** Red square frame refers to the region of staining in Vc. **(L)** Western blot showing that JNK1 is phosphorylated in the Vc after exposure to restraint stress for 14 days. *P < 0.05 vs. the control group, *n* = 4 in each group. **(M)** Effects of the intrathecal infusion of the JNK inhibitor SP600125 on masseter hyperalgesia. *P < 0.05 vs. the saline group, *n* = 4 in each group.

**Figure 3:**
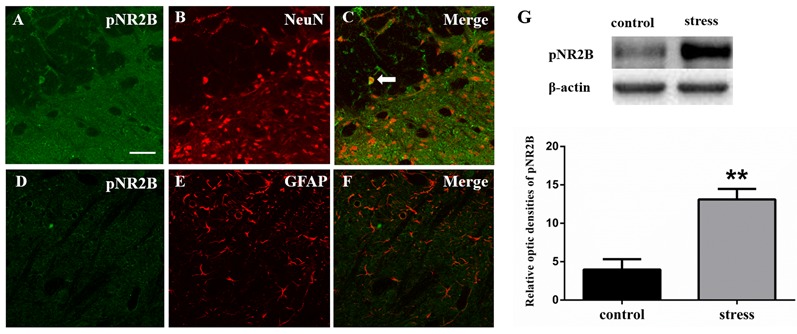
Panels **(A–F)** show the double immunofluorescence of pNR2B with NeuN or GFAP. The arrow indicates the typical double-labeled cell. Scale bars = 50 μm. **(G)** The impression of pNR2B in control and stress group. **P < 0.01 vs. the control group, *n* = 4 in each group.

**Figure 4:**
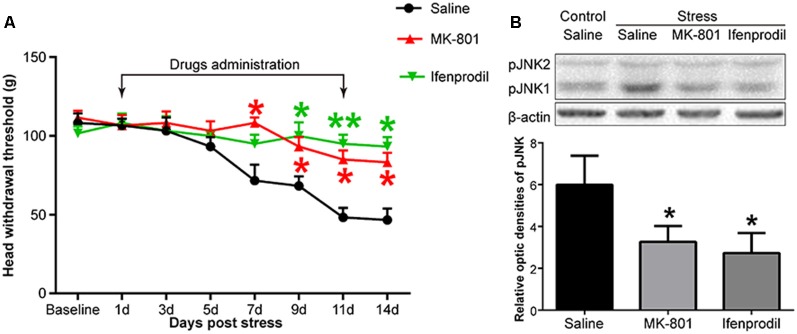
The effects of an N-methyl-D-aspartate (NMDA) receptor antagonist on restraint stress-induced masseter hyperalgesia **(A)** and JNK activation **(B)**. *P < 0.05, **P < 0.01 vs. the saline group, *n* = 4 in each group.

**Figure 5:**
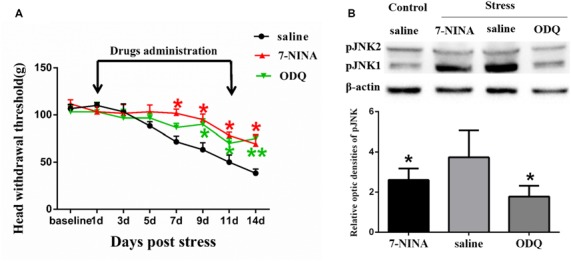
The effects of neuronal nitric oxide synthase (nNOS) selective inhibitor 7-NINA and guanylate cyclase (GC) inhibitor ODQ on restraint stress-induced masseter hyperalgesia **(A)** and JNK activation **(B)**. *P < 0.05, **P < 0.01 vs. the saline group, *n* = 4 in each group.

## Results

### Effect of Restraint Stress on Body Weight Gain, Behavior and Masseter Mechanical Sensitivity

The results demonstrated that restraint stress led to significantly lower body weight gain. Compared to the control group, the rats in the 7-days, 9-days, 11-days and 14-days stress groups (*P* < 0.05 on 7-days and 9-days, *P* < 0.01 on 11-days and 14-days, Figure 1A) exhibited significantly decreased body weight gain, whereas no significant difference was observed between the control group and the 1-day, 3-days, 5-days stress groups (*P* > 0.05, Figure 1A). In addition, restraint stress led to noticeable behavioral changes. In the open field test, the total distance traveled and the distance traveled in the center by the 7-days and 14-days stress groups were significantly shorter than those by the control group (*P* < 0.05, Figures 1B,C). Additionally, a decreased percentage of open-arm entries and a decreased open-arm retention time were observed in the 7-days and 14-days stress groups compared with the control group (*P* < 0.05, Figures 1D,E).

Furthermore, restraint stress resulted in marked mechanical hyperalgesia in the masseter muscle. The head withdrawal threshold was significantly lower in the 7-, 9-, 11- and 14-days groups than that in the control group. In the 1-day, 3-days, and 5-days stress groups, the head withdrawal threshold showed no significant compared to that of the control group (*P* > 0.05, Figure 1F).

### Astrocytic JNK Activation Contributes to Psychological Stress-induced Rat Masseter Hyperalgesia

The immunohistochemistry results showed that exposure to restraint stress for 14 days significantly increased the expression of pJNK (Figures 2A–D). The red square frame refers to the region of staining in Vc [Figure 2K, Bregma 1.60 mm (Paxinos and Watson, 2005)]. Higher magnification images (Figures 2E–J)showed double immunofluorescence of pJNK and GFAP in the two groups. The typical double-labeled astrocytes were easily seen in the stressed group whereas hardly found in the control group. We further used Western blotting to evaluate the increase in astrocytic pJNK in the spinal trigeminal nucleus 14 days after restraint stress. The results indicated that both JNK1 and JNK2 are normally expressed at high levels in the Vc. However, the expression level of pJNK-1 in the 14-days stress group was increased significantly compared with that in the control group (Figure 2L). To further confirm the role of astrocytic JNK in the Vc in masseter muscle hyperalgesia induced by restraint stress in rats, the JNK inhibitor SP600125 was intrathecally injected from the 1st day of restraint stress to the 11th day. We evaluated the change in the masseter muscle pain threshold after drug administration and observed that SP600125 significantly prevented restraint stress-induced masseter hyperalgesia in the rats from days 7 to 14 (D7 to D14; *P* < 0.05, Figure 2M).

#### Blocking NMDARs Inhibits Restraint Stress-induced Rat Masseter Hyperalgesia and JNK Activation

Like JNK, NMDARs were observed to be phosphorylated. We used Western blotting to detect the phosphorylation level of the NR2B subunit in the Vc after restraint stress. The results showed that the expression of pNR2B in the Vc in the rats exposed to restraint stress was higher than that in the control group (Figure 3G). We further evaluated the cellular localization of pNR2B in the Vc. The immunofluorescence results indicated that the pNR2B was almost co-localized with the neuronal-specific marker NeuN (Figures 3A–C). However, there was almost no pNR2B expression in astrocytes (Figures 3D–F).

To investigate whether NMDARs were involved in stress-induced masseter muscle hyperalgesia and the activation of astrocytic JNK, we administered MK-801, a non-competitive NMDAR antagonist and ifenprodil, a selective antagonist of NMDARs containing the NR2B subunit by intrathecal implantation to the stressed rats. Behavioral tests indicated that both MK-801 and ifenprodil significantly alleviated stress-induced masseter hyperalgesia (Figure 4A). The Western blotting results showed that the expression of pJNK1 was significantly increased after 14 days of restraint stress. Compared to saline administration, the intrathecal administration of MK-801 or ifenprodil effectively down-regulated the stress-induced increase in pJNK1 (Figure 4B). Taken together, these results suggested that NMDARs, specifically NR2B-containing NMDARs, are highly expressed in Vc neurons and play a pivotal role in the induction of masseter muscle hyperalgesia and astrocytic JNK activation by restraint stress in rats.

#### The Role of the nNOS-GC Pathway in Psychological Stress-induced Rat Masseter Muscle Hyperalgesia and JNK Activation

In order to further study the possible mechanism underlying NMDAR activation-induced astrocytic pJNK expression, we detected the role of nNOS-GC activation in the process of astrocytic JNK activation induced by psychological stress in rats. We administered 7-NINA, a selective inhibitor of neuronal nitric oxide synthase (nNOS) and ODQ, a selective inhibitor of guanylate cyclase (GC), and checked the change of masseter pain threshold and the expression of pJNK by Western Blotting after administration. The results indicated that intrathecal administration of both 7-NINA and ODQ not only effectively alleviated masseter hyperalgesia (Figure 5A), but also significantly inhibited the expression of pJNK induced by restraint stress (Figure 5B). The results suggested that NMDA receptor activation could increase JNK phosphorylation in astrocytes after restraint stress, which may depend on the nNOS-GC pathway.

## Discussion

Studies have shown that psychological stress is closely related to the occurrence and development of chronic orofacial pain (Huang et al., 2011; Okamoto et al., 2012). In the present study, we employed an animal model of chronic restraint stress, which avoids excessive physical stimulation and is widely used to study abnormalities in animal behavior under depressive or anxious conditions (Costa et al., 2005; Grizzell et al., 2014). The results demonstrated that repeated restraint stress led to significantly lower body weight and increased negative emotion. In the behavioral tests, the distance moved in the open-field and entry into open arm of the elevated plus-maze are important indicators to assess the anxious and depressive emotion of animals. in the present study, the animals showed decreased distance moved in the open-field and reduced time and entry into the open arm, indicating that the animals experienced increased negative emotion. Moreover, the effects were most significant 14 days after restraint stress, indicating that the chronic restraint stress applied in this study resulted in chronic psychological stress in the rats. In addition, we found that chronic restraint stress significantly decreased the mechanical pain threshold of the rats, which is consistent with previous studies. The results confirmed the correlation between psychological stress and chronic orofacial pain. Further exploring the underlying mechanisms, we noticed that a number of studies have indicated that exposure to chronic stress may cause increased masticatory muscle activity, including some symptoms of temporomandibular disorders or bruxism (Rosales et al., 2002; Tsai et al., 2002). Also in our previous study, we have found out that chronic restraint stress could cause overactivity of the masseter muscle (Song et al., 2014). Therefore, the overwork of masticatory muscle may be one of the peripheral causes of stress-induced masseter hyperalgesia. However, the central neural mechanisms underlying this correlation still need further study.

In recent years, a large number of studies (Ren, 2010) have shown that the glial cells that support neurons play a key role in the generation and maintenance of chronic pain. A previous study (Zhao et al., 2015) by our research group confirmed that astrocytes in the spinal trigeminal nucleus are involved in orofacial hyperalgesia induced by chronic psychological stress. In the nervous system, neurons are closely associated with glial cells, and the effect of glial cells on the transmission of pain information is ultimately mediated by neurons. In terms of organizational structure, nerve synapses are tightly wrapped by glial cells, which allows astrocytes and neurons to interact and affect the transmission of information. It is thought that crosstalk between neurons and astrocytes serves as a mechanism for the regulation of neuronal signals in sensory pathways (Hanani, 2005). In the central nervous system, the transmission of information between neurons is mainly dependent on nerve synapses, and the active substances contained in synapses are neurotransmitters. Glutamate, a classic neurotransmitter in the brain, plays a key role in the transmission of information. Among glutamate receptors, NMDARs are widely distributed and are one of the most important types of amino acid receptors in the central nervous system. Studies have also indicated that neurons in the Vc are activated following orofacial nociceptive stimulation and that neuronal glutamate NMDARs are involved in signal transmission (Wong et al., 2014; Takehana et al., 2017). Among all the subunits of NMDARs, NR2B subunit phosphorylation has to be demonstrated in previous studies to play a more important role than other subunits in the nociceptive transmission and neuron-astrocytic interaction (Bursztajn et al., 2004; Kato et al., 2006; Wang et al., 2011). In the present study, our results showed that restraint stress-induced the overexpression of pNR2B in the Vc. The intrathecal administration of NMDA aggravated masseter hyperalgesia induced by stress, and blocking NMDARs inhibited restraint stress-induced chronic masseter pain in the rats. These results suggest that NMDARs are involved in the induction of the masseter hyperalgesia process by chronic stress.

The MAPKs family is a family of protein kinases that plays a pivotal role in intracellular signal transduction. The JNK is one important member of MAPKs family, which has three isoforms, JNK1, JNK2 and JNK3. Various studies of chronic pain have demonstrated that JNK1/2 are mainly found in astrocytes in central neural system and play an important role in the transmission of nociceptive signals (Hansen and Malcangio, 2013; Tian et al., 2017). The results in this study also demonstrated that the expression of pJNK1 was elevated after chronic restraint stress and that a JNK inhibitor significantly prevented restraint stress-induced masseter hyperalgesia, indicating that JNK activation also contributes to stress-induced masseter hyperalgesia. In addition, previous studies have demonstrated that spinal NMDAR activation increases astrocytic JNK phosphorylation, which contributes to nerve injury-induced neuropathic pain (Wang et al., 2011), opioid-induced hyperalgesia and analgesic tolerance (Sanna et al., 2015; Deng et al., 2019). Similarly, in our study, the administration of an NMDAR agonist increased JNK expression in the Vc, and the administration of an NMDAR antagonist decreased stress-induced chronic muscle pain. These results suggest that under conditions of chronic stress, neuronal NMDAR activation activates the astrocytic JNK pathway in the Vc which contributes to masseter hyperalgesia.

Previous studies have shown that the activation of NMDA receptors leads to a massive influx of Ca^2+^ and up-regulation of the activation of some intracellular enzymes including nNOS in neurons, which were involved in the process of pain (Abe et al., 2005). The activated nNOS induced the synthesis of NO increased and studies have shown that NO plays a significant and pivotal role in the development and maintenance of hyperalgesia (Meller and Gebhart, 1993; Meller et al., 1994). Unlike most other endogenous chemical mediators, NO is a diffusible gas that readily permeates cell membranes. Based on this, we inferred that NO may diffuse into astrocytes and activate JNK. NO relies on the guanylate cyclase-cyclic guanosine monophosphate (GC-cGMP) pathway to exert its biological effects, and studies have shown that NO-GC-cGMP pathway plays an important role in spinal nociceptive transmission (Feil and Kleppisch, 2008; Schmidtko et al., 2009). In the present study, we also found that inhibitors of nNOS activation and GC inhibitor could not only inhibit the expression of pJNK in the Vc but also effectively alleviate masseter hyperalgesia induced by restraint stress, which indicated that nNOS is involved in the stress-induced JNK activation. The results suggested that NMDA receptor activation could increase JNK phosphorylation in astrocytes after restraint stress, which may depend on the nNOS-GC pathway.

It has been shown that JNK could be activated by a variety of cytokines and proinflammatory cytokines, such as TNF-α and IL-1β (Borsello et al., 2003; Gao and Ji, 2008). In the present study, we found that neuronal NMDARs in the Vc mediated JNK activation. However, it is worth noting that NMDAR antagonists do not completely block the activation of JNK. Thus, the activation of JNK in astrocytes is not entirely dependent on NMDARs, and other factors, such as cytokines, may also influence the astrocytic JNK pathway during the induction of masseter muscle hyperalgesia by restraint stress.

In summary, the present study is the first to provide evidence that the activation of astrocytic JNK in the spinal trigeminal nucleus caudalis is involved in masseter hyperalgesia induced by psychological stress. Furthermore, the activation of neuronal NMDARs, specifically NR2B-containing NMDARs, plays a key role in the phosphorylation of astrocytic JNK through reciprocal crosstalk. These findings suggest that the inhibition of intercellular communication between neurons and astrocytes may hold therapeutic promise for the treatment of orofacial pain induced by psychological stress.

## Data Availability Statement

The datasets generated during and/or analyzed during the current study are available from the corresponding author on reasonable request.

## Ethics Statement

The animal study was reviewed and approved by Animal Use and Care Committee for Research and Education of the Fourth Military Medical University (Xi’an, China).

## Author Contributions

WL, YZ and BC carried out the animal model establishment, behavioral testing, and performed intrathecal implantation, drug administration, and immunostaining assay. HZ, LM and QL carried out animal experiments, acquired the array data and performed data analysis. MZ and YC conceived the project, wrote the article, coordinated and supervised the experiments.

## Conflict of Interest

The authors declare that the research was conducted in the absence of any commercial or financial relationships that could be construed as a potential conflict of interest.
